# Development of a Circular Oriented Bioprocess for Microbial Oil Production Using Diversified Mixed Confectionery Side-Streams

**DOI:** 10.3390/foods8080300

**Published:** 2019-07-31

**Authors:** Sofia Tsakona, Aikaterini Papadaki, Nikolaos Kopsahelis, Vasiliki Kachrimanidou, Seraphim Papanikolaou, Apostolis Koutinas

**Affiliations:** 1 Department of Food Science and Human Nutrition, Agricultural University of Athens, Iera Odos 75, 11855 Athens, Greece; 2 Department of Food Science and Technology, Ionian University, 28100 Argostoli, Greece; 3 Department of Food and Nutritional Sciences, University of Reading, Reading RG6 6AD, UK

**Keywords:** food-processing, circular economy, bioprocess development, *Rhodosporidium toruloides*, microbial oil, oleic acid

## Abstract

Diversified mixed confectionery waste streams were utilized in a two-stage bioprocess to formulate a nutrient-rich fermentation media for microbial oil production. Solid-state fermentation was conducted for the production of crude enzyme consortia to be subsequently applied in hydrolytic reactions to break down starch, disaccharides, and proteins into monosaccharides, amino acids, and peptides. Crude hydrolysates were evaluated in bioconversion processes using the red yeast *Rhodosporidium toruloides* DSM 4444 both in batch and fed-batch mode. Under nitrogen-limiting conditions, during fed-batch cultures, the concentration of microbial lipids reached 16.6–17 g·L^−1^ with the intracellular content being more than 40% (w/w) in both hydrolysates applied. *R*. *toruloides* was able to metabolize mixed carbon sources without catabolite repression. The fatty acid profile of the produced lipids was altered based on the substrate employed in the bioconversion process. Microbial lipids were rich in polyunsaturated fatty acids, with oleic acid being the major fatty acid (61.7%, w/w). This study showed that mixed food side-streams could be valorized for the production of microbial oil with high unsaturation degree, pointing towards the potential to produce tailor-made lipids for specific food applications. Likewise, the proposed process conforms unequivocally to the principles of the circular economy, as the entire quantity of confectionery by-products are implemented to generate added-value compounds that will find applications in the same original industry, thus closing the loop.

## 1. Introduction

The concept of the circular economy is emerging as a worldwide strategy to transit from the current linear economy model of production and consumption to efficient resource exploitation [1,2]. Within this framework, bio-economy encompasses the holistic valorization of renewable resources towards the development of biorefinery concepts and bioprocessing schemes to produce high value-added products. Evidently, microbial lipid production constitutes a research area of paramount significance. In particular, the production of highly unsaturated microbial lipids has gained the attention of many researchers, as they could be widely employed in functional food formulations, eliciting high nutritional composition [3].

Microbial lipids are secondary metabolites synthesized by oleaginous yeasts, fungi, and algae, exhibiting an intracellular accumulation of more than 20% [4]. Nonetheless, the industrial production of microbial oil impairs numerous impediments associated with the downstream separation of intracellular lipids, carbon source, and operating cost of the fermentative process. Actually, an economic assessment of large-scale fermentation of *R*. *toruloides* using glucose resulted in an estimated unitary production cost of $3.4·kg^-1^ at an annual production capacity of 10,000 t [5]. Hence, to establish an economically feasible and sustainable process, it is imperative to fulfill certain criteria, including high productivity, the development of biorefinery concepts that generate multiple high-value end-products, or a high-end market price of the formulated product. For instance, Kopsahelis et al. [6] presented a biorefinery process suggesting the simultaneous production of protein isolate, tartrates, ethanol, polyphenols, and microbial oil from wine lees and cheese whey. Ochsenreither et al. [3] speculated that commercialization could only be achieved via the manufacture of high-value products, such as the addition of polyunsaturated fatty acids in food applications. It is also unequivocal that the cost of the initial on-set material should be negligible; thus, waste and by-products streams exhibit ideal zero or low-cost substrates for the fermentative production of microbial oil.

Among the various oleaginous microorganisms, *Rhodosporidium* species have been widely investigated for microbial oil production via the valorization of an ample range of carbon sources, including glucose, glycerol, fructose, inulin, and xylose, among them [7]. Specifically, *R*. *toruloides* exhibits advantages over other oleaginous yeasts, including rapid proliferation and high lipid accumulation on low-cost resources [8]. Leiva-Candia et al. [9] reported a microbial oil production ranging 18.1–19.2 g·L^−1^ using different sunflower meal hydrolysates, deriving from the fractionation of sunflower meal, along with crude glycerol as fermentation supplements. Likewise, *R. toruloides* was recently employed for microbial oil production, using a nutrient supplement deriving from flour-rich waste streams (FRW) and wheat milling by-products [10]. Actually, authors proposed a process to contribute towards the valorization of the massive amounts of annual waste streams that occur from confectionery manufacturing industries and bakeries, or equally as discarded, damaged, or out of date products that return on site. Current waste treatments for these streams include animal feed, composting, or disposal in landfills. The process proposed as an alternative option by Tsakona et al. employed a two-stage bioprocess, where high final lipid concentrations and conversion yields were achieved [10,11]. However, only part of the confectionery waste streams (flour-rich streams) was evaluated in the fermentation process. Besides, it is a fact that the majority of studies, dealing with microbial lipids, have been mostly applied for biodiesel and biolubricants production [3,9,12]. Nevertheless, in the context of the circular economy, some recent studies focused on the valorization of food side-streams for the development of microbial oil-based food additives, such as wax esters [13] or directed specifically on the synthesis of high oleic acid microbial lipids [14,15]. Oleic acid resulted in more than the US $350 million in revenues for 2016, whereas a constant increase is projected based on the chemical industry applications [16]. On top of that, the possibility to modulate the fractions of oleic acid in the produced lipids would enable the production of tailor-made microbial lipids for specifically targeted applications [8]. It is thus imperative that in the frame of the circular economy, food waste streams should emerge as a potential feedstock for the synthesis of microbial lipids, targeting special food applications. For instance, oleogels deriving from high unsaturated vegetable oils are desirable for the substitution of trans fat content in foods [17], which has been recently banned by U.S. Food and Drug Administration (FDA) [18].

The aim of this study was the consolidated valorization of diversified confectionary waste streams, rich in mixed carbohydrates and other micronutrients for the fermentative production of microbial lipids using the oleaginous red yeast *R. toruloides*. The present study constitutes a more integrated extension of our previous work [10,11], that implemented only part of the confectionery waste streams (flour-rich waste streams) in the investigation during the hydrolysis and fermentation process. Nonetheless, it is crucial to configure a bioprocess that will exploit the full potential of the confectionery waste streams, to generate value-added products that can be reintroduced in the food supply chain under the context of zero waste and enhanced sustainability. More specifically, the present study targeted the valorization of all different confectionery waste streams (containing sucrose, starch, and lactose, among others), which was not previously reported, towards the development of a holistic cascade bioprocess. The effect of the different confectionery side-streams was evaluated on the ability of *R*. *toruloides* to metabolize confectionary hydrolysates and shift the carbon flux towards lipid synthesis under nitrogen limitation conditions. The fatty acid profile was also evaluated, aiming to identify tailor-made food applications. Interestingly, valorization in a two-stage bioprocess of mixed confectionery waste streams, as described in the present study, entailed modifications in the composition of microbial oil. A higher degree of unsaturated lipids was obtained, advocating the potential to enhance the feasibility of the proposed scheme for further integration in existing facilities, targeting the development of high value-added products, which under the frame of the circular economy might be applied in food formulation within the initial industry.

## 2. Materials and Methods

### 2.1. Microorganisms

The fungal strain *Aspergillus awamori* 2B.361 U2/1 that was originally obtained from ABM Chemicals, Ltd. (Woodley, UK), and was kindly provided by Professor Colin Webb (University of Manchester, Manchester, UK), was employed for the production of crude enzyme consortia during solid-state fermentation (SSF). Storage, maintenance, sporulation, and inoculum preparation of the fungal strain *A. awamori* 2B.361 U2/1 have been previously reported by Tsakona et al. [11].

The oleaginous yeast strain *Rhodosporidium toruloides* DSM 4444 was used in the fermentative production of microbial oil. The strain was maintained at 4 °C, on slopes containing glucose (10 g·L^−1^), yeast extract (10 g·L^−1^), peptone (10 g·L^−1^), and agar (2%, w/v). A liquid pre-culture with the same composition was prepared as fermentation inocula.

### 2.2. Raw Materials

Wheat-milling by-products containing (w/w) 12% starch, 20% protein, and 1.1% phosphorus were employed as the solid substrate in SSF with the fungal strain *A. awamori.* Mixed food for infants (MFI), mixed confectionery waste streams (MCWS), and mixed waste streams (MWS), obtained from different categories of confectionery waste streams, were all supplied by Jotis S.A. (Athens, Greece), a Greek confectionery industry. They were involved in enzymatic hydrolytic reactions, for the formulation of fermentative substrates and subsequent microbial oil production. MFI contained (w/w) 33% starch, 17% sucrose, and 27% lactose. MCWS demonstrated a similar composition (32.3% starch, 16% sucrose, 27% lactose) along with 7% (w/w) of lipids. Lipids were extracted with n-hexane (Sigma-Aldrich, St. Louis, MO, USA) for seven days before utilization. Likewise, the composition of FRW (84.8% starch, 7.3% protein) has been previously described [10]. MWS contained a ratio of 1:1:1 MFI:MCWS:FRW and presented a final composition of (w/w) 50% starch, 11% sucrose, and 18% lactose.

### 2.3. Solid-State Fermentation and Enzymatic Hydrolytic Experiments

SSFs were conducted in 250 mL Erlenmeyer flasks for the production of crude enzyme consortia, as described by Tsakona et al. [11]. Briefly, 5 g of wheat milling by-products were added in each flask and autoclaved at 121 °C for 20 min. Subsequently, the solids were inoculated with a fungal spore suspension (2 × 10^6^ spores·mL^−1^) of *A. awamori* that was also used to adjust the moisture content of the substrate.

The fermented solids of five flasks (after 3 days of incubation at 30 °C) were suspended in 500 mL sterilized tap water and subsequently macerated using a kitchen blender. After centrifugation (9000× *g* for 10 min), individual hydrolysis of each waste stream (MFI, MCWS, or MWS) was performed by adding the supernatant in 1 L Duran bottles containing known quantities (50, 100, 150 g·L^−1^) of MFI, MCWS, or MWS. The suspension was mixed employing magnetic stirrers, and enzymatic hydrolysis was carried out at 55 °C and uncontrolled pH conditions.

At the end of enzymatic hydrolysis, the produced hydrolysates were centrifuged (9000× *g* for 10 min), and the supernatant was filter-sterilized using a 0.2 μm filter unit (Polycap TM AS, Whatman Ltd., Maidstone, UK). The pH of the hydrolysate was adjusted to 6, which is optimum for yeast growth, using 5 M NaOH.

### 2.4. Microbial Oil Fermentations

Shake flask experiments were carried out in 250 mL Erlenmeyer flasks with a working volume of 50 mL, using commercial carbon sources. Carbon sources were selected based on the composition in the hydrolysates obtained after enzymatic reactions, as indicators of the yeast performance in these hydrolysates. More specifically, commercial glucose, sucrose, fructose, and galactose were individually evaluated for yeast proliferation and microbial oil accumulation by *R*. *toruloides*. Subsequently, shake flask cultivations were performed to study the potential of *R*. *toruloides* in the hydrolysates of the three waste streams, as described in Section 2.2. All flasks were inoculated with 10% (v/v) of a 24 h exponential pre-culture of *R*. *toruloides* and incubated at 27 °C in an orbital shaker (ZHWY – 211C Series Floor Model Incubator, Zhicheng, Shanghai, China) using an agitation rate of 180 rpm. The pH value was adjusted during fermentation using 5 M NaOH when needed. Fermentations were carried out in duplicates and the respective analyses in triplicates. Data presented are the mean values of those measurements.

Bioreactor fermentations were conducted in a 3 L bioreactor (New Brunswick Scientific Co., Edison, New Jersey, USA) with a working volume of 1.5 L. The temperature and aeration were set at 27 °C and 1.5 vvm, respectively, whereas pH value was automatically adjusted to 6 with 10 M NaOH. A 10% (v/v) inoculum was applied using a 24 h exponential pre-culture. The agitation rate was controlled in the range of 150–500 rpm to maintain the dissolved oxygen concentration at 20% of saturation.

Fermentations were conducted under fed-batch mode to evaluate the utilization of MFI and MCWS hydrolysates using an optimum carbon to Free Amino Nitrogen (FAN) ratio (C/FAN) based on the results presented by Tsakona et al. [10]. Carbon was calculated based on the carbon content of sugars, whereas the FAN corresponded to the nitrogen contained in the free amino groups of amino acids and peptides in the hydrolysate. The initial FAN concentration was 292–340 mg·L^−1^, whereas initial total sugar concentration was ~50 g·L^−1^. Fed-batch fermentation strategy was achieved by the periodic addition of a concentrated solution derived from each hydrolysate (70%, w/v). The feeding solution was added in the bioreactor under aseptic conditions to sustain microbial proliferation and microbial oil synthesis. Production of total dry weight (TDW) and lipid synthesis indicated the termination of the bioprocess, which lasted up to 120 h. Samples were withdrawn to assess sugar and nitrogen consumption along with biomass, lipid, and intracellular polysaccharides (ΙPS) concentration. Residual cell weight (RCW) was determined by subtracting the produced microbial oil (g·L^−1^) from TDW (g·L^−1^).

### 2.5. Analytical Methods

The analytical methods used in this study were previously described in detail by Tsakona et al. [11]. Briefly, FAN concentration was measured with the ninhydrin colorimetric method, whereas inorganic phosphorus (IP) concentration was analyzed by the ammonium molybdate spectrophotometric method. The concentration of sugars was quantified using a High-Performance Liquid Chromatography unit (Waters 600E, Waters, Milford, MA, USA) equipped with an Aminex HPX-87H column (300 mm × 7.8 mm, Bio-Rad, Hercules, CA, USA) and a differential refractometer (RI Waters 410). Operating conditions were as follows: sample volume 20 μL; mobile phase 0.005 M H_2_SO_4_; flow rate 0.6 mL·min^−1^; column temperature 65 °C. TDW was measured by drying the yeast biomass at 105 °C for 24 h, and microbial oil (MO) was determined according to the method proposed by Folch et al. [19]. Following the disruption of dried yeast cell mass, the Folch solution, chloroform/methanol mixture at a ratio of 2:1 (v/v), was added to the cell debris. The suspension was centrifuged (9000 × *g*, 4 °C, 5 min), the solvent phase was collected, washed with 0.88% KCl (w/v), dried with anhydrous Na_2_SO_4_, and evaporated under vacuum. The fatty acid profile of microbial oil was analyzed through the production of fatty acid methyl esters (FAME) following a two-step reaction with methanol using sodium methoxide (MeONa) and HCl as catalysts. FAME was analyzed by a Gas Chromatography Fisons 8060 (Fisons Instruments, Mainz, Germany) unit equipped with a chrompack column (60 m × 0.32 mm) and an FIDdetector. Helium was used as carrier gas (2 mL·min^−1^). The analysis was carried out at 200 °C with the injector at 240 °C and the detector at 250 °C. The split ratio was 1:50, and the sample injection volume was 1 μL. Peak identification was accomplished by comparison of retention times with those of a certified reference FAME mixture (Supelco 37 Component FAME mix, Sigma-Aldrich, St. Louis, MO, USA). Fatty acid data were expressed as the area percentage of FAME.

The concentration of IPS was calculated using a modified method, described by Liang et al. [20]. Briefly, 50 mg of TDW was treated with 20 mL HCl (2.5 M). Acidification of the suspension was performed at 100 °C for 30 min followed by neutralization to pH 7 with KOH. Samples were filtered (Whatman filter paper), analyzed via the 3,5-dinitrosalicylic acid assay, and IPS were expressed as glucose equivalents, as previously described [11].

## 3. Results and Discussion

### 3.1. Enzymatic Hydrolysis of Different Mixed Confectionery Waste Streams

In a previous study, Tsakona et al. presented the production of a nutrient-rich fermentation feedstock deriving from the hydrolysis of FRW using crude enzymatic extracts obtained via SSF with *A*. *awamori* on wheat milling by-products (WMB) [11]. In this study, a similar approach was employed to hydrolyze mixed and diversified confectionery waste streams, e.g., MFI, MCWS, MWS, therefore, exploiting all potential waste streams.

The aforementioned streams contain not only starch, compared to FRW, but also other sources of carbohydrates, e.g., sucrose and lactose. Thus, the study initially targeted the hydrolysis of all carbohydrate sources into their respective monomeric sugars, to be easily assimilated by microbial entities. Likewise, the hydrolysis of proteins into adequate quantities of amino acids and peptides that can be readily metabolized during a fermentation process was also of high importance. Table 1 presents the degree of hydrolysis of starch, sucrose, and lactose content of all applied waste streams (MFI, MCWS, and MWS). Conversion of starch to glucose reached more than 95% when an initial concentration of 50 g·L^−1^ of MFI was applied. The conversion yield of sucrose to glucose and fructose reached 79.4–91.9%, whereas a similar pattern of lactose conversion to glucose and galactose was observed (75.1–89.2%) for all initial MFI solid concentrations. Starch and sucrose hydrolysis yields of MCWS and MWS (91–96.9% and 78.4–92.1%, respectively) were similar to those of MFI, while lactose conversion yields ranged from 70.1–72.3% for MCWS, and from 73.8–88.9% for MWS. The final sugar composition of the MFI and MCWS hydrolysates presented similar composition. MFI was comprised of 73.4 ± 2.1% glucose, 10.5 ± 1% fructose, and 16.1 ± 2.4% galactose, whereas MCWS contained 74.0 ± 2.7% glucose, 11.1 ± 1.3% fructose, and 14.9 ± 1.6% galactose. MWS hydrolysate contained mainly glucose (83 ± 2.7%) and lower amounts of fructose (6.6 ± 0.7%) and galactose (10.4 ± 0.9%).

The fungal strain *A. awamori* is of high industrial importance for bioprocesses; hence, it has been widely evaluated, particularly, in solid-state fermentation for the production of hydrolytic enzymes [11,21,22,23]. Smaali et al. reported on the production of extracellular thermostable invertase during submerged cultures, triggered by the addition of sucrose (1%) [23]. Bertolin et al. studied the production of glucoamylase via SSF of *A*. *awamori* on wheat bran [24]. Grape pomace was employed as the sole substrate for the production of cellulase, xylanase, and pectinase with *A. awamori* 2B.361 U2/1 [25]. The authors reported cellulase activity up to 9.6 ± 0.76 IU/gds during the first 24 h of fermentation. Cellulases comprise three distinct categories, e.g., endo-1,4-β-glucanase, exo-1,4-β-D-glucanase, and β-glucosidase, cleaving the β-1.4 linkages in cellulose. McGhee et al. studied the cultivation of *A. awamori* on wheat bran to produce α-galactosidase and invertase [26]. The proliferation of two different *Aspergillus* strains, including *A*. *awamori*, on several agro-industrial by-products, were evaluated for the production of α-galactosidase [27]. Tsakona et al. [10,11] employed the same process to formulate fermentation supplements from FRW. In that case, the authors reported on the enzymatic hydrolysis of starch to glucose. Hence, the results obtained from our study are in accordance with previous studies, indicating that the crude enzymatic extracts produced after SSF contain the essential hydrolytic enzymes to break down polysaccharides and oligosaccharides present in MFI and MCWS to generate a rich supplement for bioconversion processes (Table 1). Therefore, the novelty of the present work focuses on the enzymatic hydrolysis of mixed confectionery waste streams containing all sugars to generate the respective monomers. This will allow for the development of a more consolidated process to exploit all waste streams, under the frame of developing bioconversion processes that can integrate and fit into the circular economy concept.

### 3.2. Shake Flask Fermentations for Microbial Oil Production

As demonstrated in Table 1, the high degree of hydrolysis in MFI entailed the formulation of hydrolysates rich in glucose, fructose, and galactose. The similar composition was obtained in the hydrolysates of MCWS and MWS (Table 1). Thus, the next step would target the consumption of these sugars sources for the proliferation and microbial oil synthesis by the strain *R*. *toruloides*. Experiments were initiated by the fermentation of pure commercial sources of each sugar using shake flasks, and the results are presented in Table 2. It can be easily seen that after 140 h, substrate consumption was terminated yielding 36–43% of intracellular microbial oil. These experiments were the preliminary step to ensure that *R*. *toruloides* could consume glucose, sucrose, fructose, and galactose, thus the potential to utilize the hydrolysates obtained from MFI, MCWS, and MSW.

**Figure 1:**
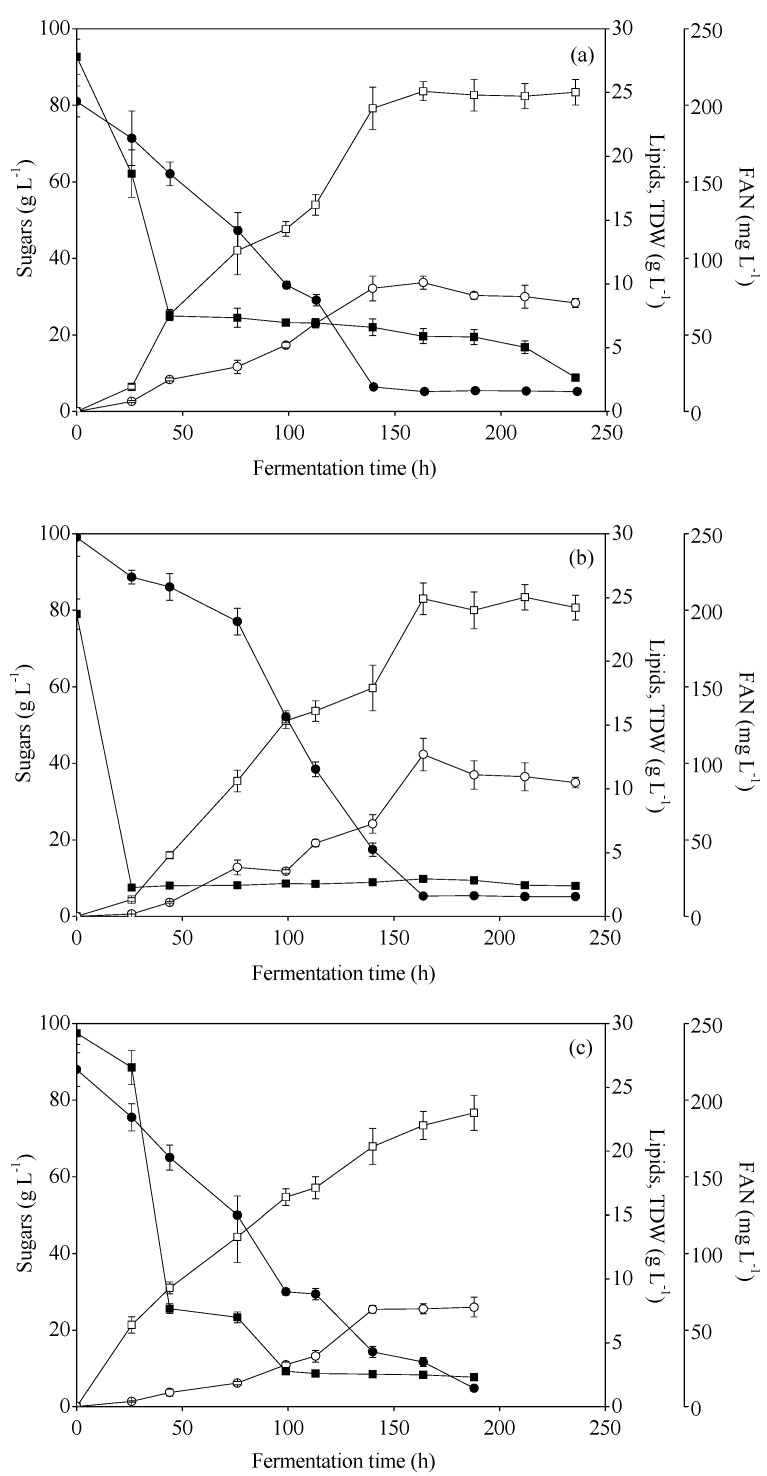
Concentration of sugars (●), free amino nitrogen (FAN) (■) and production of total dry weight (TDW) (□) and microbial lipids (○), during shake flask cultures of *R. toruloides* on (**a**) mixed food for infant (MFI) hydrolysates, (**b**) mixed confectionery waste streams (MCWS) hydrolysates, and (**c**) mixed waste streams (MWS) hydrolysates.

Hence, the next step employed the utilization of these hydrolysates as the sole fermentation supplements for lipid synthesis. Figure 1 illustrates the consumption of total sugars along with TDW and lipid production expressed in g·L^−1^, using the three different hydrolysates (a: MFI, b: MCWS, c: MWS). In all cases, the initial total sugar concentration was in the range of 80–100 g·L^−1^, whereas FAN concentration ranged from 197–243 mg·L^−1^. Lipid synthesis started approximately after 44 h of fermentation, triggered by nitrogen depletion in the medium (Figure 1). One of the targets of these experiments was to also identify the better performing substrate as a fermentation feedstock. The hydrolysates from MFI and MCWS resulted in a maximum 42.6% and 52.9% intracellular content of lipids, respectively, whereas in the case of MWS hydrolysate, the microbial oil content did not exceed 37.5%. Τhe highest oil productivity was achieved in the case of MCWS (0.077 g·L^−1^·h^−1^), followed by MFI (0.069 g·L^−1^·h^−1^) and MWS (0.054 g·L^−1^·h^−1^). Interestingly, *R. toruloides* presented higher specific growth rate (0.28–0.29 h^−1^) when MFI and MCWS were employed as substrates, as compared to MWS (0.12 h^−1^). Notably, the consumption rate of total sugars was lower in the first hours of fermentation when MCWS hydrolysate was tested compared to MFI hydrolysate. More specifically, the consumption rate was 0.43 and 0.31 g·L^−1^·h^−1^ in the case of MFI and MCWS, respectively, at the early phase of the fermentation (44 h). Still, in all used substrates, the obtained conversion yield of lipids to TDW was more than 0.3 (g g^−1^).

The strain *R*. *toruloides* constitutes an industrially important strain for the production of microbial lipids, carotenoids, and various enzymes [8]. Xu et al. reported the ample range of carbohydrates strains belonging to *Rhodotorula* species could consume, including glucose, fructose, xylose, arabinose, sucrose, starch, inulin, and glycerol [7]. In particular, for *R*. *toruloides*, the ability to synthesize lipids via cultivation on cassava starch, glucose, xylose, glycerol, and distillery wastewater has been well established [7].

So far, co-cultivation of *R*. *toruloides* was studied in the viewpoint of exploiting the hydrolysates obtained from lignocellulosic biomass, targeting either lipids or even carotenoids production [28,29]. Contradictive results have been reported based on the combination of substrates and the specificity of strains. For instance, Matsakas et al. demonstrated the parallel consumption of glucose and fructose using the strain *R*. *toruloides* CCT 0783 on dried sorghum stalks, achieving a final lipid concentration of 13.77 g·L^−1^ [28]. On the other hand, the combination of glucose, xylose, and arabinose resulted in the utilization of xylose after glucose depletion, whereby arabinose was not metabolized [30]. The authors employed detoxified sugarcane bagasse hydrolysates, reaching a lipid production equal to 12.3 ± 0.5. Similarly, Martins et al. evaluated a carob pulp syrup for carotenoids production during fed-batch fermentation of *R*. *toruloides* NCYC 921, where they demonstrated that glucose was first metabolized, whereas sucrose was not consumed, thus indicating a growth-limiting factor [29]. Bommareddy et al. presented a reconstructed metabolic model, using genomic and proteomic approach, on cell growth and synthesis of triglycerides (TAG) on glycerol, glucose xylose, arabinose, and various combinations [31]. Fermentation of glycerol and glucose presented catabolite repression phenomena whereby causing the sequential consumption of glycerol after glucose.

Table 3 shows the fatty acid profile of *R. toruloides* lipids generated during shake flask cultures on commercial sugars (glucose, sucrose, fructose, and galactose). The major fatty acids were oleic (C18:1) and palmitic (C16:0), whereas lower quantities of stearic (C18:0) and linoleic (C18:2) acids were observed. Oleic and palmitic acids corresponded to more than 75% (w/w) of the total amount of fatty acids synthesized by *R*. *toruloides*. This is in accordance with the results reported by Tchakouteu et al. [32], whereby oleic and palmitic acid reached 76.7% of the total fraction of lipids produced. Slininger et al. reported that cultivation of *R*. *toruloides* NRRL Y-1091 using lignocellulosic hydrolysate resulted in 72.9% of oleic and palmitic [33], whereas in another study, *R*. *toruloides* Y4 reached 66.9%, after 134 h of fed-batch fermentation [34].

Similarly, in our previous study using FRW hydrolysate during fed-batch bioreactor experiments, almost 80% of the total concentration of fatty acid corresponded to oleic and palmitic acid [10].

### 3.3. Fed-Batch Bioreactor Cultures Using MFI and MCWS Hydrolysates

One of the major targets of this study was to identify the potential of MFI, MCWS, and MWS hydrolysates as sole fermentation substrates for lipids production. Following the results from shake flask cultures, the next step employed the evaluation of the best performing substrates (MFI and MCSW) in bioreactor cultures.

Nitrogen limitation is known to induce de novo synthesis of lipids in oleaginous strains as it is a secondary activity, occurring while the carbon source is in excess [35,36]. Deficiency of nitrogen in the fermentation broth leads to the low activity of isocitrate dehydrogenase; thus, metabolic flux is directed to lipid overproduction [35,37]. This case is induced at high carbon to nitrogen (C/N) ratios, whereby biomass production is hindered, and the carbon source is channeled into lipogenesis [37].

**Figure 2:**
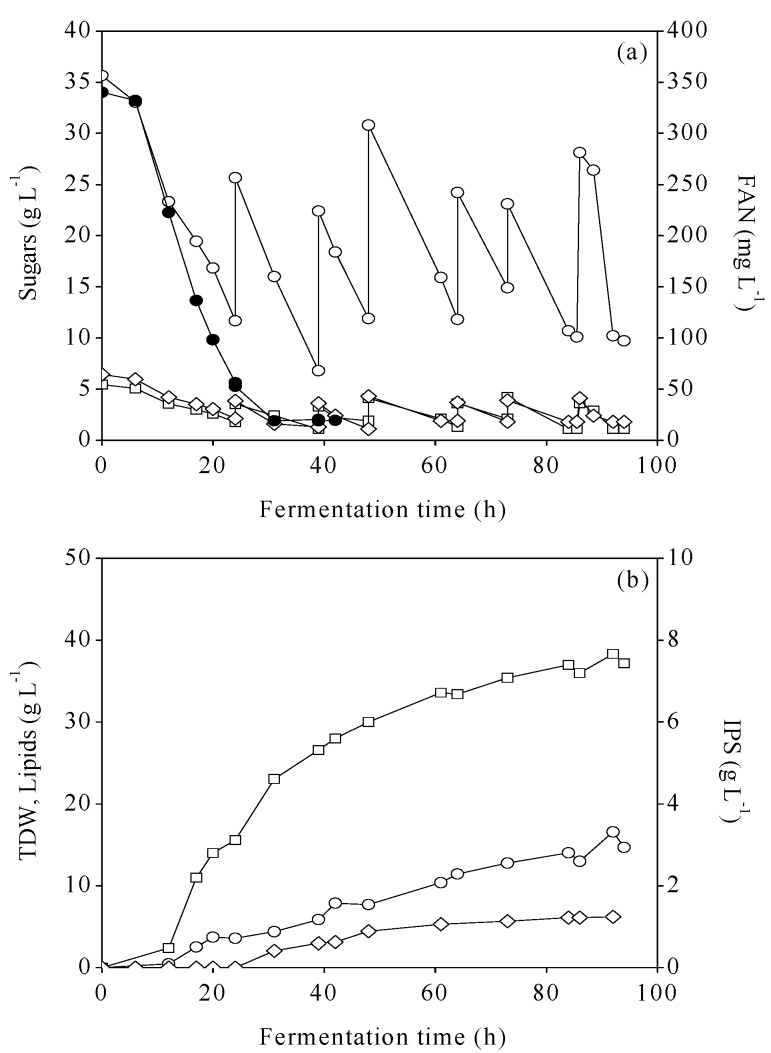
(**a**) Concentration of glucose (○), fructose (□), galactose (◊), free amino nitrogen (FAN) (●) and (**b**) production of total dry weight (TDW) (□), microbial lipids (○), intracellular polysaccharides (IPS) (◊) during fed-batch bioreactor fermentations of *R. toruloides* on mixed food for infants (MFI) hydrolysates.

In this study, the initial C/N ratio was selected following our previous work with *R*. *toruloides* and FRW hydrolysates [10], hence adjusted to ~70–80 g·g^−1^. Figure 2a presents the time course consumption of total sugars (glucose, fructose, and galactose), along with FAN utilization, whereas in Figure 2b, the production of TDW, microbial lipids, and IPS are presented. Initial total sugar concentration was ~50 g·L^−1^, with glucose being the main sugar, whereas fructose and galactose were present in low concentrations (5.4 and 6.4 g·L^−1^, respectively). Intermittent additions of the feeding solution were performed, when sugars’ concentration was lower than 20 g·L^−1^. Maximal concentration of microbial lipids was observed after 92 h of fermentation (16.6 g·L^−1^) with an intracellular content of 43.3% (w/w) and a productivity of 0.18 ·L^−1^·h^−1^.

**Figure 3:**
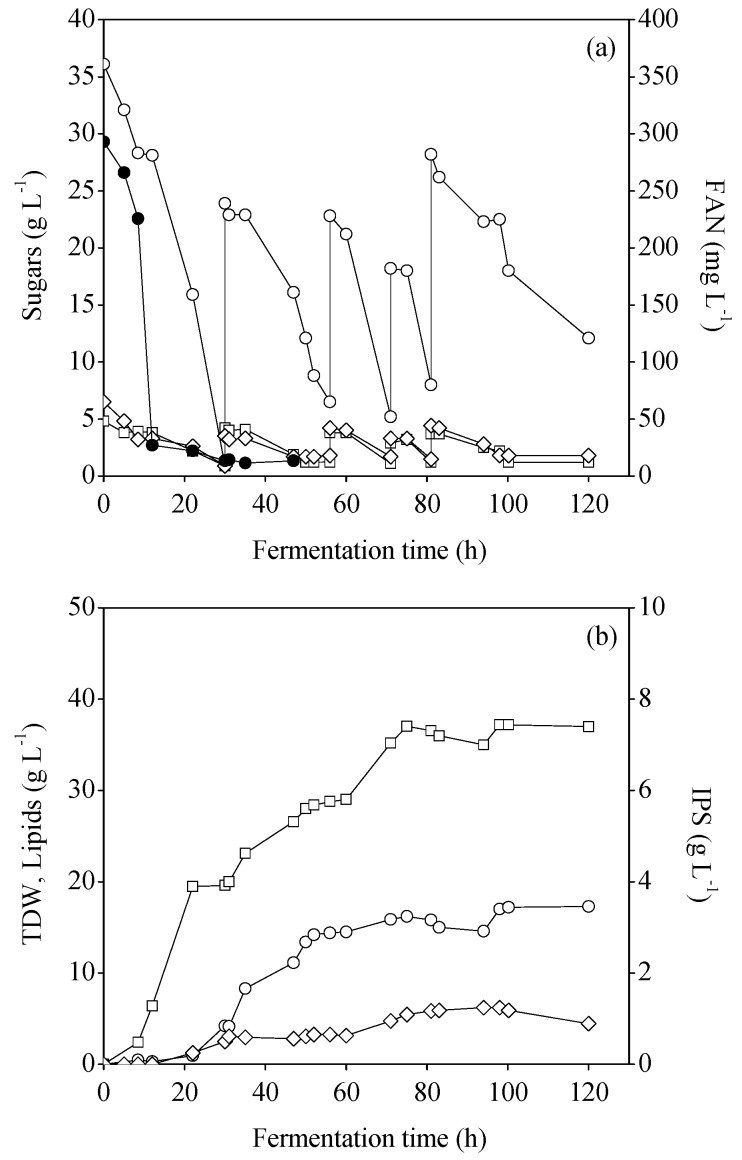
(**a**) Concentration of glucose (○), fructose (□), galactose (◊), free amino nitrogen (FAN) (●) and (**b**) production of total dry weight (TDW) (□), microbial lipids (○), intracellular polysaccharides (IPS) (◊) during fed-batch bioreactor fermentations of *R. toruloides* on mixed confectionery waste streams (MCWS) hydrolysates.

Similarly, Figure 3 depicts the results obtained when MCWS hydrolysate was evaluated by applying a fed-batch strategy. In this case, lipid production reached 17 g·L^−1^ after 98 h, resulting in productivity of 0.17 g·L^−1^·h^−1^. The lipid content was 45.6% (w/w).

It is easily observed that when both MFI and MCWS hydrolysates were employed, FAN depletion from the media channeled carbon flux to lipid overproduction. This observation was also confirmed by the fact that RCW remained constant until the end of fermentation. It is also interesting to note that consumption of glucose, fructose, and galactose occurred at the same time in both cases, without indicating carbon catabolite repression phenomena in the presence of glucose. These results are in agreement with the results reported by Matsakas et al., where glucose and fructose were equally consumed [28].

Studies on the simultaneous consumption of carbon sources have been recently initiated, and significant work is undertaken on transcriptomic and proteomic levels to understand the metabolic network [31,38]. Bommareddy et al. presented a metabolic model of *R*. *toruloides* by using different carbon sources. When glucose was compared with glycerol during bioreactor fermentations, increased biomass was noted in the case of glucose, regardless of the final production of lipids, which was almost equal in both cases [31]. Following flux distribution analysis, the authors showed that when glucose comprised the sole carbon and energy source, 63% of NADPH derived from the pentose phosphate (PP) pathway, complimented by the cytosolic malic enzyme [31]. On the other hand, when glycerol alone was employed, NADPH was supplied by the cytosolic malic enzyme, indicating a reduction in PP pathway. Similarly, using the pentoses xylose and arabinose, high PP pathway was observed to meet the demand for NADPH uptake [31]. The same authors, further performed transcriptomics to investigate gene expression during the combined fermentation of glucose with glycerol [38]. Significant findings concerning the upregulation and downregulation of genes were presented, particularly during the phase of nitrogen starvation [38].

Combination of glucose with glycerol entailed a diauxic growth; however, glycerol addition improved lipid synthesis. Wiebe et al. evaluated C5 and C6 sugars, particularly glucose, xylose, arabinose, and their combinations, during batch and fed-batch cultures. They stated that lipid production in the mixture was lower compared to individual application of glucose or xylose, regardless of the proportions of glucose and xylose in the mixture [39]. In another study, Easterling et al. used the oleaginous strain *R*. *glutinis* during fermentation of dextrose, xylose, and glycerol and their mixtures, demonstrating that mix substrate cultivations resulted in increased biomass production compared to individual carbon sources [40]. On top of that, the strain *R*. *toruloides* has been previously shown to produce IPS [11,32], which was also observed in this study (~1.2 g·L^−1^). Analysis of IPS by HPLC at the end of the fermentation demonstrated that they were primarily (>60%) comprised of mannose and glucose and to a lesser extent of galactose and fructose.

Table 4 presents the fatty acid profile of the microbial lipids produced during fed-batch experiments using MFI and MCWS hydrolysates. As expected, oleic (C18:1), stearic (C18:0), palmitic (C16:0), and linoleic (C18:2) were the major fatty acids identified. Compared to FRW employed in our previous work [10], the proportions of the individual fatty acid were modified, an observation deriving probably by the different composition of the substrate. More specifically, oleic acid (C18:1) remained the major fatty acid produced; however, it was increased from approximately 52.5–61.4% when MFI and MCWS were used. When the latter substrates were employed, the fractions of palmitic acid (C16:0) and linoleic (C18:2) were significantly reduced, whereby the fractions of stearic acid (C18:0) increased almost 2-fold compared to FRW hydrolysates. Similarly, the fraction of palmitic acid was decreased when MFI and MCWS were used in fed-batch experiments compared to shake flask cultures, where individual carbon sources were used. On the other hand, oleic acid was increased when MFI and MCWS were applied for lipid bioconversion. The differences found in fatty acid composition when different substrates (FRW, MFI, and MCWS) were employed may be attributed to their different sugar composition. In fact, FRW contains only glucose, whereas MFI and MCWS contain also fructose and galactose. The effect of substrate composition on the fatty acid profile of *R. toruloides* microbial oil has been highlighted also by previous studies [7,14]. Further research studies focused on proteomics and transcriptomics have demonstrated that gene expression and substrate specificity have a key role in lipid synthesis [41]. Specifically, Fillet et al. mentioned that the final chain length of the fatty acid relates to the substrate preference of the elongase 3-ketoacyl-CoA [42]. Furthermore, Zhu et al. pointed that the yield of polyunsaturated fatty acids depends on the number of an acyl carrier protein (ACP), which is part of the fatty acid synthase system, exhibiting a significant role in the chain-elongation process [36].

The applied feeding strategy during fed-batch cultures, oxygen saturation, and primarily carbon source constitute key factors on the final profile analysis of TAGs produced. The effect of feeding strategy and dissolved oxygen was beyond the scope of the current study; thus, the emphasis was given on the carbon source. When FRW hydrolysates were used [10], unsaturated content was found to be 61.8%, a proportion that increased in both cases where MFI and MCWS were employed (72.1 and 69.7%, respectively). It is generally accepted that de novo synthesis of lipids in *R*. *toruloides* results mainly in unsaturated fatty acids (e.g., oleic and linoleic acid) [7]. Fei et al. used corn stover hydrolysates and reported that oleic acid (C18:1) was the major fatty acid produced in all applied strategies, followed by palmitic (C16:0) [49]. Wiebe et al. reported that stearic, linoleic, and palmitic fractions were affected in the presence of mixed carbon sources compared to pure glucose fermentation experiments, whereby xylose and arabinose addition induced an increase in C16:0 and C18:2 fatty acids [39]. Patel et al. noted that palmitic acid was not detected when glucose and fructose were used as fermentation sources with *R*. *kratochvilovae* HIMPA1 [50]. The presence of glucose and fructose led to increased monounsaturated fatty acids, whereas sucrose increased polyunsaturated fatty acids. Interestingly, Bommareddy et al. [31] showed a content of 57% in saturated fatty acids when glucose alone was employed, whereas, during shake flask cultures in our study, the corresponding amount reached approximately 37.6% (Table 3). This could be attributed to the conditions during fermentation (bioreactor compared to shaking flasks), thus indicating future research. Zeng et al. undertook the utilization of food waste hydrolysates containing glucose as the major carbon source during flask experiments, demonstrating that oleic (75.8%) was the predominant fatty acid, followed by palmitic, linoleic, and lower quantities of stearic [51]. As previously noted, oxygen in the fermentation medium is also crucial for the formulation of lipid fractions. Minkevich et al. also stated the variation between saturated and unsaturated fatty acid profile with oxygen limitation [52], whereby Bommareddy et al. pointed the production of saturated fatty acids following un-controlled oxygen supply [31]. On the other hand, the provision of glycerol in the fermentation media resulted in increased saturated fractions. For instance, a combination of crude glycerol and sunflower meal hydrolysates [9] resulted in 43.1% of saturated fatty acids, whereas Signori et al. reported 42.2–42.4% of saturated content when pure and crude glycerol were used, respectively [53].

Previous studies on microbial oil production through bioconversion processes focus primarily to evaluate the lipids for biodiesel production through transesterification processes [54,55]. However, under the viewpoint of bio-economy transition, emphasis should be also given to novel applications of microbial lipids for the production of lipid-based products with improved quality and specific applications [13]. The production of food products with a limited amount of saturated fat has emerged to be of paramount importance for food industries during the last years. For instance, high unsaturated vegetable oils (i.e., soybean oil) are preferred for the production of oleogels, which are considered a healthier substitute for trans and saturated fats in food products [17]. Bharathiraja et al. reviewed the application of microbial lipids in food formulations, stating how they could replace plant-derived lipids (e.g., cocoa butter, palm oil) and further utilized as stabilizing and thickening agents, emulsifiers, and water-binding compounds [56]. Equally, given the high content of oleic acid in the produced lipids, it could be also used as a substitute for cocoa butter, based on the high content of unsaturated fatty acids.

Apart from texture, fatty acids can also regulate the aroma and flavor of specific types of food. Likewise, fatty acids can be implemented in pharmaceutical applications, and more specifically, to formulate fortified foods and/or beverages with polyunsaturated fatty acids, entailing possible health effects [56]. Concerning microbial lipids from *R*. *toruloides*, Papadaki et al. conducted a study on the valorization of yeast lipid derivatives to formulate bio-based wax esters through a two-stage biocatalysis reaction [57]. Implementation of molasses as fermentation supplement led to the production of 8.1 g·L^−1^ microbial oil, containing mainly oleic acid (51%), palmitic acid, and stearic acid. The generated oleogels, using olive oil as the base oil, successfully simulated commercial margarine, thus demonstrating their potential for future applications in spreadable fat-products [57]. It should be stressed that the results obtained in the present study highlight that the valorization of the whole confectionery waste streams potential through the proposed process enhance the feasibility for further integration in existing facilities. Likewise, the proposed scheme leads to the production of lipids with a higher degree of unsaturation, a result that could be exploited under the frame of the circular economy towards the development of high value-added products for targeted food formulations within the initial industry.

## 4. Conclusions

The potential of generating nutrient-rich fermentation supplements deriving from diversified confectionery waste streams has been well established. All the evaluated hydrolysates performed well both in shake flask cultures and fed-batch bioreactor cultivations using *R*. *toruloides* to produce microbial lipids. Implementation of mixed substrates containing glucose, fructose, and galactose did not affect the consumption rates, however, resulted in modifications in the fractions of fatty acids. The possibility to produce tailor-made microbial lipid fractions for the production of lipid-based products was also presented. A consolidated bioprocess previously developed to valorize mixed confectionery waste streams could be further expanded to also target future food applications of microbial lipids under the context of the bio-economy era.

## Figures and Tables

**Table 1:** Degree of hydrolysis (%, w/w) of starch, sucrose, and lactose during hydrolysis experiments, using varying initial solid concentrations of mixed food for infants (MFI), mixed confectionery waste streams (MCWS), and mixed waste streams (MWS) hydrolysates.

Waste Stream Concentration	Composition	MFI	MCWS	MWS
50 g·L^−1^	Starch	95.6 ± 0.42	96.9 ± 0.89	93.6 ± 0.89
	Sucrose	91.9 ± 1.48	90.9 ± 1.96	92.1 ± 0.67
	Lactose	89.2 ± 0.92	72.3 ± 2.75	88.9 ± 1.28
100 g·L^−1^	Starch	93.4 ± 1.06	94.5 ± 0.85	91.3 ± 1.62
	Sucrose	83.9 ± 2.26	88.9 ± 1.02	83.6 ± 1.34
	Lactose	81.8 ± 3.54	71.3 ± 0.98	78.1 ± 0.71
150 g·L^−1^	Starch	91 ± 1.63	93.6 ± 0.56	91 ± 0.99
	Sucrose	79.4 ± 2.19	84.2 ± 1.93	78.4 ± 1.59
	Lactose	75.1 ± 2.83	70.1 ± 1.63	73.8 ± 2.59

**Table 2:** Consumption of substrate, along with the production of total dry weight (TDW), microbial oil (MO), and intra-cellular oil content during the cultivation of *R. toruloides* using commercial glucose, sucrose, fructose, and galactose at 30 g·L^−1^.

Substrate	Fermentation Time (h)	Consumed Substrate (g·L^−1^)	TDW (g·L^−1^)	MO (g·L^−1^)	Oil Content (%, w/w)
**Glucose**	141	30 ± 0.3	9.4 ± 0.1	3.7 ± 0.3	39.3 ± 2.4
**Sucrose**	147	30 ± 0.2	10.5 ± 0.3	4.6 ± 0.3	43.8 ± 1.5
**Fructose**	147	29.4 ± 0.9	9.7 ± 0.4	3.5 ± 0.1	36.1 ± 0.9
**Galactose**	147	29.6 ± 0.6	9.1 ± 0.1	3.6 ± 0.1	39.6 ± 1.9

**Table 3:** Fatty acid composition of lipids produced during shake flask cultures of *R. toruloides* on commercial sugars (glucose, sucrose, fructose, galactose).

Fermentation Time (h)	C14:0	C16:0	^Δ9^ C16:1	C18:0	^Δ9^ C18:1	^Δ9,12^ C18:2	^Δ9,12,15^ C18:3
**Glucose**							
60	1.8	34	0.3	7.6	43.2	8.4	4.13
92	1.3	27.4	0.8	8	46.9	9.5	3.5
140	0.9	24.5	0.8	5.9	51.5	12.3	4
**Sucrose**							
23	1.9	34.7	-	7.9	42.8	8.6	4.1
103	1.2	27.1	0.8	8.5	48.9	9.7	3.7
147	0.9	24.9	0.8	6.1	50.7	12.4	4.1
**Fructose**							
45	1.6	28.1	1.1	7.5	46.8	10.9	2.7
103	1.4	27.9	0.7	7.9	48.7	9.5	3.6
147	0.9	24.5	0.9	5.7	51.5	12.3	3.7
**Galactose**							
24	1.2	26.2	0.9	6.8	45.3	11.1	2.7
105	1.6	27.5	0.9	7.5	49.7	8.9	3.8
140	0.9	24.9	0.9	6.8	50.3	11.3	4.6

**Table 4:** Fatty acid composition of plant-derived oils compared with the composition of the microbial lipids produced during fed-batch bioreactor cultures of *R. toruloides* using the hydrolysates from mixed food for infants (MFI) and mixed confectionery waste streams (MCWS).

Oil Source	Fatty Acids (%)							Reference
	C14:0	C16:0	^Δ9^ C16:1	C18:0	^Δ9^ C18:1	^Δ9,12^ C18:2	^Δ9,12,15^ C18:3	
Soybean	–	6–10	0.1	2–5	20–24.9	50–60	4.3–11	[43,44,45,46,47]
Rapeseed	–	2.8–14		0.9–2	13.6–64.1	11.8–26	7.5–13.2	[43,44,45,46,47]
Cottonseed	–	27–28.7	–	0.9–2	13–18	51–58	8	[43,44,45,46,47]
Sunflower	–	4.6–6.4	0.1	2.9–3.7	17–62.8	27.5–74	0.1–0.2	[43,44,45,46,47]
Palm oil	0.7	36.7–44	0.1	5–6.6	3–46.1	8.6–11	0.3	[43,44,45,46,47]
Olive oil	<0.1	7.5–20	0.3–3.5	0.5–5	55–83	3.5–21.0	≤1.0	[48]
	**Microbial lipids from bioreactor cultures**							
**FRW** ^a^	1.5	28.7	0.6	7.5	50.3	9.5	1.4	[10]
**MFI**	1.4	10.3	0.7	14.5	61.2	5.3	0.4	This study
**MCWS**	0.9	15.2	0.9	13.8	61.7	6.1	0.1	

^a^ FRW: flour-rich waste.

## Abbreviations

FRW—Flour-rich waste streams, SSF—Solid-state fermentation, MFI—Mixed food for infants, MCWS—Mixed confectionery waste streams, MWS—Mixed waste streams, FAN—Free amino nitrogen, TDW—Total dry weight, RCW—Residual cell weight, IP—Inorganic phosphorus, MO—Microbial oil, FAME—Fatty acid methyl esters, IPS—Intracellular polysaccharides, WMB—Wheat milling by-products, TAG—Triglycerides.
